# Disturbances of the hypothalamic-pituitary-adrenal axis and plasma electrolytes during experimental sepsis

**DOI:** 10.1186/2110-5820-1-53

**Published:** 2011-12-30

**Authors:** Michael A Flierl, Daniel Rittirsch, Sebastian Weckbach, Markus Huber-Lang, Kyros Ipaktchi, Peter A Ward, Philip F Stahel

**Affiliations:** 1 Department of Orthopaedics, School of Medicine, University of Colorado, Denver Health Medical Center, Denver, CO, 80204, USA; 2 Department of Trauma Surgery, University Hospital Zurich, Zurich, Switzerland; 3 Department of Trauma, Hand-, Plastic-, and Reconstructive Surgery, University Hospital Ulm, Ulm, 89075, Germany; 4 Department of Pathology, University of Michigan Medical School, Ann Arbor, MI, 48109, USA; 5 Department of Neurosurgery, University of Colorado, School of Medicine, Denver Health Medical Center, Denver, CO, 80204, USA

## Abstract

**Background:**

Sepsis continues to be a poorly understood syndrome with a high mortality rate. While we are beginning to decipher the intricate interplay of the inflammatory response during sepsis, the precise regulation of the hypothalamic-pituitary-adrenal (HPA) axis and its impact on electrolyte homeostasis during sepsis remains incompletely understood.

**Methods:**

Sepsis was induced in adult male Sprague-Dawley rats by cecal ligation and puncture (CLP). Plasma samples were obtained as a function of time (6-48 hrs) after CLP and compared with healthy animals (neg ctrl). Samples were analyzed for adrenocorticotropin (ACTH), corticosterone, and aldosterone levels, as well as concentrations of sodium (Na^+^), potassium (K^+^), chloride (Cl^-^), and magnesium (Mg^2+^).

**Results:**

ACTH levels were found to be significantly reduced 6-24 hrs after CLP in comparison to baseline levels and displayed gradual recovery during the later course (24-48 hrs) of sepsis. Plasma corticosterone concentrations exhibited a bell-shaped response, peaking between 6 and 12 hrs followed by rapid decline and concentrations below negative control levels 48 hrs after injury. Aldosterone levels in septic animals were continuously elevated between 6 and 48 hrs. Whereas plasma Na^+ ^levels were found to be persistently elevated following CLP, levels of K^+^, Cl^- ^and Mg^2+ ^were significantly reduced as a function of time and gradually recovered during the later course of sepsis.

**Conclusions:**

CLP-induced sepsis resulted in dynamic changes of ACTH, corticosterone, and aldosterone levels. In addition, electrolyte levels showed significant disturbances after CLP. These electrolyte perturbations might be evoked by a downstream effect or a dysfunctional HPA-axis response during sepsis and contribute to severe complications during sepsis.

## Introduction

Sepsis remains an enigmatic, poorly understood disease [1]. Disturbingly, there has been a rapid increase of hospitalization and mortality rates between 1993 and 2003 [2], making sepsis the tenth leading cause of death in the United States [3]. Despite numerous encouraging preclinical results for new therapeutic approaches to sepsis, a successful transfer from "bench to bedside" has yet to be achieved [4-10]. Various randomized clinical trials investigating anti-inflammatory strategies have failed to show any survival improvement [11]. To date, the only clearly beneficial treatment for the septic patient is early goal-directed therapy [12].

During an immune response, the central nervous and immune system extensively communicate with each other [13]. The major pathways involved in this cross-talk are the hypothalamic-pituitary-adrenal (HPA) axis and the autonomic nervous system [13-15]. Immune mediators and cytokines released by the innate immune system trigger regional neural and systemic neuroendocrine responses, both of which seek to return the system to a homeostatic state [13]. Steroids are hormonal key players antagonizing and down-regulating inflammation [16]. However, during sepsis, the HPA axis may become severely dysfunctional [13,17]. In humans, sepsis is known to induce an abnormal pituitary response, resulting in profound hormonal changes in ACTH, growth hormone, vasopressin, cortisol, mineralocorticoids, and thyroid hormones [18,19].

Vermes and colleagues studied regulatory mechanisms of the hypothalamo-pituitary-adrenal system in critically ill patients and found elevated plasma levels of cortisol and ACTH in septic and trauma patients [20]. Whereas plasma concentrations of cortisol remained elevated for 8 days, plasma ACTH decreased between days 3 to 5. Plasma levels of endothelin-1 and atrial natriuretic hormone were significantly elevated during the entire observation period. Accordingly, the authors speculated that the high endothelin-1 level may exert a positive drive on the adrenocortical level, whereas elevated high ANH level may inhibit the HPA axis during critical illness. Thus, critically ill patients can develop metabolic alkalosis, hyperreninemic hyperaldosteronism, and hypokalemia.

Many reports about the HPA axis during sepsis represent "snapshot" measurements of HPA axis function, rather than a methodical assessment as a function of time, and are mostly performed following ACTH stimulation tests [21]. Moreover, most of the literature focuses on the anti-inflammatory properties of adrenal-derived glucocorticoids. A thorough evaluation of the downstream events triggered by a dysfunctional aldosterone response, such as severely altered electrolyte homeostasis, has yet to be investigated.

In the present study, we sought to investigate the pituitary-adrenal-electrolyte axis in experimental CLP-induced sepsis in a systematic approach. In parallel with the existing human studies, we hypothesized that the HPA axis might be severely dysfunctional with subsequent severe electrolyte disturbances.

## Methods

### Experimental CLP model

All procedures were performed in accordance with the National Institutes of Health guidelines and University Committee on Use and Care of Animals, University of Michigan (UCUCA approval #8575). Specific pathogen-free, adult male Sprague-Dawley rats (Harlan Inc., Indianapolis, IN) weighing 300-350 g were used in all experiments. Sepsis was induced by the CLP procedure as previously described [22,23]. In brief, rats were anesthetized with isoflurane (3%, oxygen flow 3L O_2_/min). After abdominal midline incision, the cecum was exposed, ligated, and punctured through and through with an 18-gauge needle, and a small portion of feces was expressed to ensure patency of the punctures. After repositioning of the bowel, the abdomen was closed in layers using 4-0 surgical sutures (Ethicon Inc., Somerville, NJ) and metallic clips. Sham animals underwent the same procedure except for ligation and puncture of the cecum. Before and after the surgery, animals had unrestricted access to food and water.

### Plasma isolation

Rat whole blood was collected into syringes containing anticoagulant citrate dextrose (ACD; Baxter, Deerfield, IL) in a 9:1 ratio by puncture of the inferior vena cava as a function of time after CLP surgery. Samples were centrifuged (2,500 rpm, 10 min, 4°C) and plasma was obtained and immediately stored at -80°C until further analysis. Because ACTH and corticosterone are released in a circadian fashion, peaking between 8-9 PM in night-active rodents [24], all samples were obtained at 8 PM to avoid baseline fluctuations.

### ELISA analysis of rat ACTH, rat corticosterone, and rat aldosterone

Levels of ACTH, corticosterone, and aldosterone were determined by using commercially available ELISA kits (ACTH: Phoenix Pharmaceuticals, Belmont, CA; Corticosterone: Diagnostic Systems, Webster, TX; Aldosterone: Cayman Chemical, Ann Arbor, MI) according to the manufacturer's instructions. The ACTH ELISA had a minimum detectable concentration of 0.08 ng/ml and a range from 0-25 ng/ml. The Corticosterone EIA had a minimum detectable concentration of 1.6 (range, 6-2000) ng/ml, and the Aldosterone assay had a minimum detectable concentration of 21 (range, 7.8-1000) pg/ml. Assays were performed strictly according to the manufacturer's instructions.

### Assessment of plasma electrolyte levels

After plasma isolation, samples were run on a standardized chemistry analyzer, which applies plasma directly to individually wrapped slides ("Vettest 8008" chemistry analyzer, IDEXX Laboratories, Inc., Westbrook, ME). Any needed dilutions were performed with 0.9% NaCl. Electrolyte levels were then directly evaluated by the companion standardized IDEXX VetLyte analyzer (IDEXX Laboratories, Inc.).

### Statistical analysis

All values are expressed as means ± SEM. Data were analyzed with a one-way ANOVA, and individual group means were then compared with the Tukey multiple comparison test. Differences were considered statistically significant at *P *< 0.05.

## Results

### Experimental sepsis induces severe depression of systemic ACTH levels

**Figure 1:**
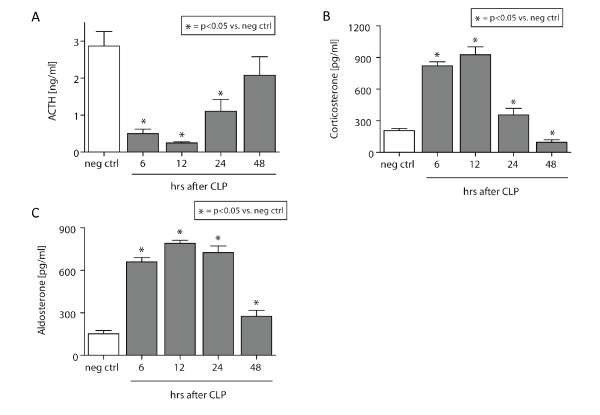
**Hormonal changes of the HPA-axis following experimental sepsis**. **(A) **Plasma levels obtained from healthy rats (neg ctrl) or septic littermates (6-48 hrs after CLP). **(B) **Plasma samples from septic animals were obtained 6-48 hrs after CLP and compared with healthy rats. **(C) **Aldosterone concentrations were assessed as a function of time in septic vs. healthy rats. All samples were analyzed by ELISA. *n *= 5-10 per experimental condition.

Plasma was isolated from whole blood from healthy negative control animals (neg ctrl) or CLP rats, and subjected to quantitative ELISA analysis for ACTH. As shown in Figure 1A, there was a substantial reduction in ACTH levels in plasma after CLP. Whereas negative control levels of ACTH ranged at 3 ng/ml, CLP induced a highly significant reduction of plasma ACTH levels between 6 hrs and 24 hrs after induction of sepsis, with a nadir 12 hrs after CLP surgery (~0.3 ng/ml). After 48 hrs, ACTH levels gradually returned to levels found in negative controls.

### Transient hypercortisolism with subsequent corticosterone deficiency in plasma during CLP

Corticosterone represents the major glucocorticoid of rodents [25]. Plasma samples were assessed for corticosterone as a function of time after CLP and revealed a bell-shaped response (Figure 1B). Initially, hypercorticosolism was observed during the early course of experimental sepsis (6-24 hrs), with peak levels (~900 pg/ml) reaching more than threefold elevated concentrations compared with neg ctrl levels (~220 pg/ml). During the late course of CLP-induced sepsis (48 hrs), rats presented with significantly reduced corticosterone levels of 150 pg/ml in comparison to neg ctrl animals.

### Hyperaldosteronism after CLP-induced sepsis

Plasma levels of aldosterone were determined in neg ctrl animals or septic rats, as a function of time after CLP by ELISA analysis. During the complete observation time following CLP (6-48 hrs), aldosterone levels were found to be significantly elevated after CLP-induced sepsis, peaking between 6-24 hrs, where levels were found to be greater than fourfold increased compared with the neg ctrl group (Figure 1C).

### Electrolyte disturbances during sepsis

**Figure 2:**
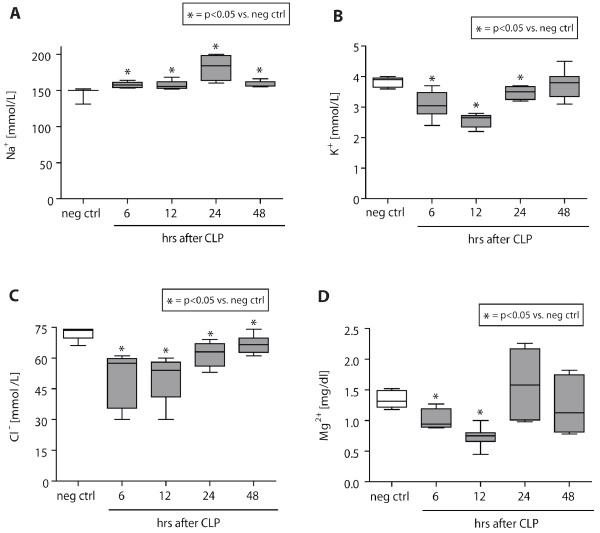
**Electrolyte levels during experimental sepsis**. Plasma samples were obtained and evaluated with a standardized chemistry analyzer for electrolyte levels as a function of time after CLP. Healthy animals served as negative controls. Electrolytes assessed include sodium **(A)**, potassium **(B)**, chloride **(C)**, and magnesium **(D)**. *n *= 5-10 per experimental condition.

Plasma samples were obtained from healthy, negative control animals and animals subjected to CLP and evaluated for sodium (Na^+^), potassium (K^+^), chloride (Cl^-^), and magnesium (Mg^2+^) levels (Figure 2). Na^+ ^levels were found to be significantly elevated during 6-48 hrs after CLP, with a peak after 24 hrs (175 mmol/L vs. 150 mmol/L in neg ctrl animals) (A). In contrast, K^+ ^concentrations in septic rats were significantly reduced 6-24 hrs after CLP compared with neg ctrl rats (4 mmol/L vs. 2.7 mmol/L) and returned to negative control levels 48 hrs after injury (B). During the entire course of observation, septic rats displayed significantly reduced levels of Cl^- ^compared with healthy animals, reaching a nadir 12 hrs after CLP (75 mmol/L vs. 52 mmol/L) (C). Plasma levels of Mg^2+ ^initially decreased significantly 6-12 hrs after CLP (0.75 mg/dl) and returned to negative control levels (1.3 mg/dl) 24 hrs after induction of experimental sepsis (D).

## Discussion

Despite advanced, aggressive ICU management of the septic patient, lethality rates remain at 40-60% for patients in septic shock [26]. Multiple promising experimental approaches have failed to show a successful therapeutic translation into humans [10]. Initially, these failures might have been attributable to an overly simplified theoretical understanding of sepsis as a merely proinflammatory state [27]. Subsequently, this notion has been challenged [28], requiring a careful reconsideration and revisiting our current concept of the pathophysiology behind the sepsis syndrome [1,29].

Triggered by the septic inflammatory response, endogenous glucocorticoids are being released, presumably in an attempt to modulate and counterbalance the synthesis and release of inflammatory mediators on a cellular level [13]. However, vascular and ischemic damage, inflammation, and apoptosis within the HPA-axis itself [30] can severely impair the HPA-axis and prompt adrenal insufficiency, a well-described complication during sepsis [31]. Glucocorticoid insufficiency may result in an imbalanced T-cell response with uncontrolled systemic inflammation [32]. If unrecognized and untreated, impaired HPA axis function may result in a lethal outcome [33,34]. Thus, current recommendations advocate the use of corticosteroids in critically ill patients with adrenal insufficiency [35]. However, in a large clinical trial, high-dose corticosteroids significantly increased morbidity and is therefore considered obsolete for the treatment of severe sepsis and septic shock [36]. As a result, current recommendations suggest the use of moderate doses of corticosteroids in septic patients [35]. Nonetheless, glucocorticoid substitution in the septic patient remains a matter of lively debate [37], as previous studies used different methodology (± ACTH test or not), and study group characteristics (sepsis vs. septic shock). This is likely due to the fact that sepsis-induced adrenal insufficiency seems to be highly multifactorial and is associated with a very complex and poorly understood pathophysiology.

In the present study, we investigated the function of the HPA-axis as a function of time after experimental, CLP-induced sepsis. Focusing on ACTH, corticosterone, and the mineralocorticoid aldosterone, we assessed the dynamic changes of these HPA-axis hormones. In addition, we sought to assess the changes in plasma electrolyte homeostasis following CLP. We describe bell-shaped plasma levels of both, corticosterone and aldosterone (Figures 1B, C). In septic patients, initial hypercortisolism during the early stages of sepsis also has been described and is usually followed by cortisol insufficiency [18]. Although there is evidence that, in septic patients, a clear dissociation between ACTH and cortisol levels exists [38], our present data imply that, in rodents, the physiologic feed-back loops of the HPA-axis may be intact during the early course of experimental sepsis (Figures 1A, B). However, during the later course of CLP, there is likely breakdown of the blood-brain barrier with ensuing pituitary dysfunction, resulting in dissociation of the HPA axis [19]. Such a breakdown of physiologic barriers will result in extravasation of inflammatory markers and cells, bacteria, ultimately disrupting the classic HPA feedback mechanisms. Previous reports have described sepsis-induced plasma alterations of ACTH and corticosterone concentrations only during the late stages of sepsis [39,40]. Clinical studies describe persistent hypercortisolism despite low ACTH levels in septic humans, which may reflect breakdown of feedback mechanisms in humans and/or neuronal or mediator-induced hormonal changes. Our discordant findings may represent one of the disconnects observed between rodents and men [41]. For instance, rats are known to produce glucocorticoids in extra-adrenal organs as well, which may result in a different steroid response in experimental sepsis [42].

Sepsis and septic shock are important risk factors for acute renal failure (ARF) and represent the most important trigger for ARF in the ICU [43,44]. Twenty percent of patients with severe sepsis and 50% of patients with septic shock have been shown exhibit ARF [45]. In addition, ARF has previously been described in rats following CLP [46]. In the current study, we found severe plasma electrolyte disturbances. Animals displayed significant hypernatremia following CLP (Figure 2A). Sepsis has been shown recently to be associated with hypernatremia, which seemed to serve as an independent predictor for higher mortality in these patients [47]. It remains to be determined whether the alterations of especially sodium and potassium levels observed in our study are indeed a result of ARF or rather represent a downstream effect of the significantly increased aldosterone levels found in septic animals (Figure 1C). In the present study, septic rats also exhibited severe hypomagnesemia (Figure 2D), which also has been described in up to 65% of ICU patients [48,49]. More importantly, magnesium deficiency in the ICU is associated with prolonged hospitalization [50], two- to threefold higher mortality rates, and the development of sepsis [51]. An experimental study revealed that magnesium administration reduced the severity of septic encephalopathy [52], which represents a serious complication of sepsis in the ICU with high morbidity and mortality [53]. In this study, animals treated with magnesium sulfate displayed attenuated blood-brain barrier breakdown and reduced brain edema compared with vehicle-treated septic littermates [52].

Our study has several shortcomings. First, the animal model did not use any resuscitative measure or antibiotic coverage but only studied the natural course of disease. This limits the transferability of our findings into the human setting even more in addition to the traditional, well-described disconnect between humans and rodents [41]. Second, we did not provide a true hormonal time-course for sham controls. However, plasma samples were obtained exactly at the same time of the day for all experiments to account for the circadian rhythm of HPA axis hormones. Thus, it was assumed that sham animals maintained an intact HPA axis. In addition, our study is merely descriptive and fails to unravel any molecular mechanisms involved in the breakdown of the HPA axis in sepsis. Complement C5a may play an important role in the pathogenesis of the HPA disturbance [19]. Nevertheless, our study provides novel insights into the sepsis-induced disturbance of the HPA axis.

## Conclusions

Sepsis continues to be a poorly understood syndrome. To be able to develop novel therapeutic approaches, we have to broaden our understanding about its intricate and multimodal pathophysiology. Disturbances of the HPA axis and its hormones may contribute to several severe complications encountered in the septic patient. Thus, consecutive studies need to be designed to further unravel the complex cross-talk between the HPA axis and the immune response.

## Competing interests

The authors declare that they have no competing interests.

## Authors' contributions

MAF, PFS, MHL, and PAW designed the study and supervised the experiments. MAF and DR performed all experiments. MAF, PFS, SJM, and WRS analyzed the data and drafted the manuscript. All authors revised the manuscript for important scientific content, read, and approved the final manuscript.
